# Identification of bacteria on the surface of clinically infected and non-infected prosthetic hip joints removed during revision arthroplasties by 16S rRNA gene sequencing and by microbiological culture

**DOI:** 10.1186/ar2201

**Published:** 2007-05-14

**Authors:** Kate E Dempsey, Marcello P Riggio, Alan Lennon, Victoria E Hannah, Gordon Ramage, David Allan, Jeremy Bagg

**Affiliations:** 1 Infection and Immunity Research Group, Level 9, Glasgow Dental Hospital & School, 378 Sauchiehall Street, Glasgow G2 3JZ, UK; 2 The Queen Elizabeth National Spinal Injuries Unit, Scotland, South Glasgow University Hospitals Division, Southern General Hospital, 1345 Govan Road, Glasgow G51 4TF, UK

## Abstract

It has been postulated that bacteria attached to the surface of prosthetic hip joints can cause localised inflammation, resulting in failure of the replacement joint. However, diagnosis of infection is difficult with traditional microbiological culture methods, and evidence exists that highly fastidious or non-cultivable organisms have a role in implant infections. The purpose of this study was to use culture and culture-independent methods to detect the bacteria present on the surface of prosthetic hip joints removed during revision arthroplasties. Ten consecutive revisions were performed by two surgeons, which were all clinically and radiologically loose. Five of the hip replacement revision surgeries were performed because of clinical infections and five because of aseptic loosening. Preoperative and perioperative specimens were obtained from each patient and subjected to routine microbiological culture. The prostheses removed from each patient were subjected to mild ultrasonication to dislodge adherent bacteria, followed by aerobic and anaerobic microbiological culture. Bacterial DNA was extracted from each sonicate and the 16S rRNA gene was amplified with the universal primer pair 27f/1387r. All 10 specimens were positive for the presence of bacteria by both culture and PCR. PCR products were then cloned, organised into groups by RFLP analysis and one clone from each group was sequenced. Bacteria were identified by comparison of the 16S rRNA gene sequences obtained with those deposited in public access sequence databases. A total of 512 clones were analysed by RFLP analysis, of which 118 were sequenced. Culture methods identified species from the genera *Leifsonia *(54.3%), *Staphylococcus *(21.7%), *Proteus *(8.7%), *Brevundimonas *(6.5%), *Salibacillus *(4.3%), *Methylobacterium *(2.2%) and *Zimmermannella *(2.2%). Molecular detection methods identified a more diverse microflora. The predominant genus detected was *Lysobacter*, representing 312 (60.9%) of 512 clones analysed. In all, 28 phylotypes were identified: *Lysobacter enzymogenes *was the most abundant phylotype (31.4%), followed by *Lysobacter *sp. C3 (28.3%), gamma proteobacterium N4-7 (6.6%), *Methylobacterium *SM4 (4.7%) and *Staphylococcus epidermidis *(4.7%); 36 clones (7.0%) represented uncultivable phylotypes. We conclude that a diverse range of bacterial species are found within biofilms on the surface of clinically infected and non-infected prosthetic hip joints removed during revision arthroplasties.

## Introduction

Prosthetic joints are a major advance in the practice of modern medicine and have revolutionised the life of many patients. At least 50,000 total hip replacements are performed each year in the UK [1]. The incidence of hip replacements worldwide is expected to increase from 1.66 million in 1990 to 6.26 million in 2050 and, in the European Union, an increase from 414,000 to 972,000 cases per annum is expected over the next 50 years [2]. Unfortunately the risk of infection is a significant problem, resulting in high rates of morbidity and creating a massive economic burden.

Prosthetic joint infections of total hip arthroplasties (THAs) reportedly occur with an incidence of 1.5% for the primary THA and 3.2% for the revision THA [3]. However, one group demonstrated that up to 15% of hip replacements in their study were infected [4]. Several of these infections are characterised by biofilms, adherent communities of bacteria attached to the prosthetic hip joint components that are resistant to antibiotic challenge and host immunity [5].

One of the major problems in accurately determining the infection rate is the difficulty in isolating, by traditional culture methods, the bacteria from the surface of the prosthetic hip joint [6]. The reasons for this include strongly adherent bacteria in the biofilm and the presence of antibiotic-containing cement. One method developed to improve the microbial yield was to place the hip prosthesis directly into an anaerobic jar after surgical removal, followed by mild ultrasonication of the prosthesis to remove adherent microbes and processing of the specimens within an anaerobic cabinet [7]. Another reason for the low yield of microbial growth may be that the joint is infected with highly fastidious and non-cultivable, or viable but non-cultivable, bacteria that cannot be isolated with standard techniques. This problem can be overcome by the use of molecular techniques to detect the microbial DNA from bacteria present on the prosthesis. All culture-independent, molecular-based studies that have investigated the microflora in a wide range of environmental and clinical samples have identified a greater diversity of bacteria than culture methods alone [8,9]. More specifically, this was found to be true in one study that identified bacteria associated with failed prosthetic hip joints [10]. Using PCR, these workers detected bacteria in 72% of the prosthetic hip joints removed during revision arthroplasty, whereas there was only a 22% detection rate by conventional culture. In addition, these workers were able to reveal bacteria directly by immunofluorescence confocal microscopy of sonicates from previously uncultured specimens. Overall, this indicated that the incidence of prosthetic hip joint infection is grossly underestimated by conventional culture methods.

The purpose of this study was to identify bacteria within the biofilms on the surface of clinically infected and non-infected prosthetic hip joints by using both 16S rRNA-based molecular detection methods and conventional microbiological culture. Ten prosthetic hip joints were analysed for the presence of bacteria by PCR amplification, cloning, and sequence analysis of bacterial 16S rRNA genes. The results obtained were compared with data obtained from aerobic and anaerobic microbiological culture of the same samples. The clinical interest of this study is the presence of any organism on the prosthetic hip joints and the role, if any, that they have in initiating, prolonging or activating simultaneous or subsequent clinical infections.

## Materials and methods

### Selection of patients

Patients undergoing prosthetic hip joint revisions were recruited from those attending the Department of Orthopaedic Surgery at the Southern General Hospital, Glasgow. Each patient gave written informed consent to participate in the study. Ethical approval was obtained from the Ethics Committee of the Southern General Hospital, Glasgow.

### Clinical samples and clinical data

Prosthetic hip joints were collected by a surgical team wearing body exhaust suits in an operating theatre with a clean-air enclosure. Ten prosthetic hip joint implants were retrieved by two different surgeons from patients undergoing revision hip surgery at the Southern General Hospital, Glasgow, during a 4-month period. Demographic and clinical data for the 10 patients are shown in Table 1. All 10 cases were clinically and radiologically loose, with a varying risk of infection shown by the raised levels of the infection markers C-reactive protein and erythrocyte sedimentation rate. Taken together with postoperative progress and results of conventional bacteriology, this suggested that five prosthetic hip joint implants were removed as a result of clinical infection and five as a result of aseptic loosening of the prosthesis. On removal, the femoral and acetabular cup components of the prosthetic hip joint were placed into sterile plastic bags and immediately transported to Glasgow Dental Hospital and School for analysis. Several preoperative and perioperative samples were also taken from each patient, including hip joint aspirate, capsular fluid, acetabular membrane, femoral membrane and (in certain cases) pus, which were sent to the bacteriology laboratory at the Southern General Hospital, Glasgow, for analysis. During these revision operations no prophylactic antibiotics were administered until the bacteriology samples had been obtained and the prosthesis had been removed. The antibiotic-loaded cement used at each primary revision was cefuroxime with gentamicin.

**Table 1:** Clinical details of the 10 patients studied

Patient no.	Sex	Age	CRP (mg/l)	ESR (mm/h)	Hb (g/l)	WCC (× 10^9 ^g/l)	Clinical diagnosis	Bacteriology results	Duration prosthesis in place (months)
1	M	73	< 5	10	117	6.1	Aseptic loosening	No growth	178
2	M	69	< 3	ND	150	6.5	Aseptic loosening	No growth	48
3	M	61	61	49	130	10.6	Infected	Coagulase-negative *Staphylococcus *(CF, AM, FM)	5
4	F	56	36	ND	134	6.9	Aseptic loosening	No growth	79
5	M	65	36	14	142	6.7	Infected	No growth	55
6	F	66	< 10	14	148	7.7	Infected	Coagulase-negative *Staphylococcus *(CF, AM, FM)	n.d.
7	F	49	45	60	120	8.8	Infected	*Proteus mirabilis *(AM, FM)	n.d.
8	M	59	80	30	169	4.2	Aseptic loosening	No growth	120
9	M	62	ND	ND	119	3.9	Aseptic loosening	No growth	n.d.
10	M	57	131	ND	106	10.7	Infected	No growth	n.d.

CRP, C-reactive protein (reference range 0 to 6 mg/l); ESR, erythrocyte sedimentation rate (reference range 1 to 13 mm/h (male), 1 to 20 mm/h (female)); Hb, haemoglobin (reference range 130 to 170 g/l (male), 120 to 150 g/l (female)); WCC, white cell count (reference range 4.0 to 10.0 ng/l); AM, acetabular membrane; CF, capsular fluid; n.d., not determined; FM, femoral membrane.

### Processing of preoperative and perioperative samples

With some minor amendments, preoperative and perioperative samples were processed as described previously [6]. In brief, samples were disrupted by vigorous agitation with sterile glass beads in sterile diluent. Aliquots of the tissue suspension were inoculated onto blood agar and chocolate blood agar plates for incubation in a CO_2 _incubator and onto fastidious anaerobe agar (FAA) containing blood for anaerobic incubation. Gram staining was performed with a portion of the sample, and the rest of the sample was inoculated into fastidious anaerobe broth. Plates were examined daily for 7 days and the broths were subcultured at 5 days, or sooner if turbid.

### Processing of prosthetic hip joint components

The femoral and acetabular components of the prosthetic hip joint were processed separately to remove adherent bacteria. The removal of bacteria from the hip joint components was performed with a Fisherbrand FB11021 sonicating water bath (Fisher Scientific, Loughborough, UK) in a class II microbiological safety cabinet. All equipment including the water bath, plasticware, pipettes and plastic bags were sterilised by ultraviolet irradiation. Each hip joint component was sealed in a sterile plastic bag to which 40 or 20 ml of sterile water was added for the femoral component or acetabular cup component, respectively. The sealed bags were then put into the sonicating water bath for 5 minutes at 350 Hz. This process has previously been shown not to affect bacterial viability negatively [10]. Sonicate (10 ml) from each component was pooled and subjected to microbiological culture as described below. The remaining volume of sonicate for each prosthetic hip component was then transferred to a sterile tube and centrifuged at 1,000 *g *for 20 minutes. The supernatant was discarded and the bacterial pellet was resuspended in 0.5 ml of sterile water, pooled for each component and stored at – 80°C until required for molecular analysis.

### Microbiological culture

Each sonicate was centrifuged for 5 minutes at 2,500 r.p.m.; the pellet was suspended in 1 ml of phosphate-buffered saline and 10-fold serial dilutions to 10^-6 ^were prepared. All dilutions (from undiluted to 10^-6^) were spiral plated onto both Columbia agar containing 7.5% (v/v) defibrinated horse blood and FAA (BioConnections, Wetherby, UK) containing 7.5% (v/v) defibrinated horse blood. Dilutions (from undiluted to 10^-3^) were also spiral plated onto skimmed milk agar, nutrient agar and CY-agar plates. Columbia blood agar plates were incubated in 5% CO_2 _at 37°C, and FAA plates were incubated at 37°C in an anaerobic chamber with an atmosphere of 85% N_2_, 10% CO_2 _and 5% H_2_. Skimmed milk agar, nutrient agar and CY-agar plates were incubated in 5% CO_2 _at 30°C. Plates were examined after 1, 3 and 7 days, and morphologically distinct colonies were subcultured to obtain pure cultures. Isolates were identified by 16S rRNA gene sequencing as described below.

### DNA extraction

A crude DNA lysate of bacterial DNA from the prosthesis sonicate was prepared. Samples were mechanically disrupted with 1.0 mm glass beads (Thistle Scientific Ltd., Glasgow, UK) and a Mini-BeadBeater (Stratech Scientific, Newmarket, UK). These were homogenised three times for 30 seconds at 48 Hz, with cooling on ice between homogenisations. An aliquot of the homogenate was then used for DNA extraction. To 100 μl of homogenate was added 3 μl of achromopeptidase (20 U/μl in 10 mM Tris-HCl, 1 mM EDTA, pH 7.0), followed by incubation at 56°C for 1 hour. Samples were boiled for 10 minutes, debris was removed by centrifugation and the supernatant was retained for PCR analysis. DNA was stored at – 20°C until required. DNA was extracted from bacterial isolates by the same method.

### Polymerase chain reaction (PCR)

The primers used for amplification targeted conserved regions of the 16S rRNA gene and were designed to amplify DNA from most bacterial species. The primers used were 5' -AGA GTT TGA TCM TGG CTC AG-3' (27f; *Escherichia coli *nucleotides 8–27) and 5' -GGG CGG WGT GTA CAA GGC-3' (1387r; *E. coli *nucleotides 1,387-1,404; MWG Biotech, Milton Keynes, UK), where M = C or A and W = A or T, and give an expected amplification product of about 1,400 base pairs [11]. All PCR reactions were conducted in a total volume of 50 μl, comprising 5 μl of extracted bacterial DNA and 45 μl of reaction mixture containing 1 × PCR buffer (10 mM Tris-HCl pH 9.0, 50 mM KCl, 1.5 mM MgCl_2_, 0.1% Triton X-100), 1.0 U *Taq *DNA polymerase (Promega, Southampton, UK), 0.2 mM dNTPs (GE Healthcare, Little Chalfont, UK) and each primer at a concentration of 0.2 μM. PCR was performed in an OmniGene thermal cycler (Hybaid, Teddington, UK). The PCR cycling conditions comprised an initial denaturation step at 94°C for 5 minutes, followed by 35 cycles of denaturation at 94°C for 1 minute, annealing at 58°C for 1 minute and extension at 72°C for 1.5 minutes, and finally an extension step at 72°C for 10 minutes.

### PCR quality control

When performing PCR, stringent procedures were employed to prevent contamination, as described previously [12]. Negative and positive controls were included with each batch of samples being analysed. The positive control comprised a standard PCR reaction mixture containing 10 ng of *E. coli *genomic DNA instead of sample; the negative control contained sterile water instead of sample. Each PCR product (10 μl) was subjected to electrophoresis on a 2% agarose gel, and amplified DNA was detected by staining with ethidium bromide (0.5 μg/ml) and examination under ultraviolet illumination.

### Cloning of 16S rRNA PCR products

PCR products were cloned into pGEM-T Easy cloning vector by using the pGEM-T Easy Vector System I Kit (Promega), in accordance with the manufacturer's instructions.

### PCR amplification of 16S rRNA gene inserts

After cloning of the 16S rRNA gene products amplified by PCR for each sample, 50 clones from each generated library were randomly selected. The 16S rRNA gene insert from each clone was amplified by PCR with the use of the primer pair 5' -GCT ATT ACG CCA GCT GGC GAA AGG GGG ATG TG-3' (M13FAP) and 5' -CCC CAG GCT TTA CAC TTT ATG CTT CCG GCA CG-3' (M13RAP). The M13FAP binding site is located 32 base pairs upstream of the M13 forward primer binding site, and the M13RAP binding site is located 39 base pairs downstream of the M13 reverse primer binding site, in the pGEM-T Easy vector.

### Restriction enzyme analysis

Selected clones from the libraries generated from the 10 prosthetic hip samples were subjected to restriction enzyme analysis with *Rsa*I and *Mnl*I. About 0.5 μg of each PCR product was digested at 37°C in a total volume of 15 μl with 2.0 U of *Mnl*I (Helena Biosciences, Sunderland, UK) or 2.0 U of *Rsa*I (Promega) for 3 hours. Restriction fragments were detected by agarose gel electrophoresis as described above. For each library, clones were initially sorted into distinct restriction fragment length polymorphism (RFLP) groups on the basis of restriction profiles obtained with *Rsa*I. Further discrimination was obtained by digestion of clones with *Mnl*I, a restriction enzyme that is highly effective at generating unique bacterial 16S rRNA fingerprints. This resulted in the identification of additional distinct RFLP groups.

### DNA sequencing

The 16S rRNA gene of a single, representative clone from each RFLP group identified by restriction enzyme analysis was sequenced. The resultant PCR products from the recombinant clones were purified with the QIAquick PCR Purification Kit (QIAGEN, Crawley, UK) in accordance with the manufacturer's instructions. Sequencing reactions were performed with the Fermentas Life Sciences CycleReader™ Auto DNA Sequencing Kit (Helena Biosciences) and IRD800-labelled M13 universal (- 21); (5' -TGT AAA ACG ACG GCC ACT-3') or 16S rRNA 357F (5' -CTC CTA CGG GAG GCA GCA G-3') primer on a Primus96 DNA thermal cycler (MWG Biotech, Milton Keynes, UK) with the use of the following cycling parameters: an initial denaturation step at 92°C for 2 minutes, followed by 30 cycles of denaturation at 94°C for 30 seconds, annealing at 52°C for 30 seconds and extension at 72°C for 1 minute. Direct sequencing of bacterial isolates was performed with the IRD800-labelled 357F primer, whereas sequencing of recombinant clones was carried out with IRD800-labelled M13 universal (- 21) primer. Formamide loading dye (6 μl) was added to each reaction mixture after thermal cycling. Each denatured sequencing reaction mixture (1.5 μl) was run on a LI-COR Gene ReadIR 4200S automated DNA sequencing system (LI-COR Biosciences UK Ltd, Cambridge, UK) in accordance with the manufacturer's instructions.

### 16S rRNA gene sequence analysis

Sequence data were compiled with LI-COR Base ImagIR 4.0 software, converted to FASTA format and compared with 16S rRNA gene sequences from public sequence databases (GenBank and EMBL) using the advanced gapped BLAST program, version 2.1 [13]. Clone sequences possessing at least 98% identity with a sequence in the GenBank/EMBL databases were considered to be that species.

## Results

### Culture-dependent methods

Bacteriology results for the preoperative and perioperative samples (hip joint aspirate, capsular fluid, acetabular membrane, femoral membrane and, in certain cases, pus) taken from each of the 10 patients are shown in Table 1. Bacteria were identified in at least one of these samples in only 3 of the 10 patients. Coagulase-negative *Staphylococcus *was identified in the capsular fluid, acetabular and femoral membranes of two different cases (patients 3 and 6). *Proteus mirabilis *was identified in the acetabular and femoral membranes of patient 7. The three cases from which bacteria were identified were all clinically infected. No bacterial growth was observed for the other seven cases analysed.

Bacteria were isolated from all five clinically infected and five clinically non-infected prosthetic hip joints. From the 10 prosthetic hip joints analysed, a total of 46 bacterial isolates were obtained and identified by 16S rRNA gene sequencing.

Table 2 shows the isolates obtained from the 10 prosthetic hip joints by culture and identified by 16S rRNA gene sequencing; they are grouped according to genera. Species belonging to the genus *Leifsonia *were the most prevalent, accounting for over half of the isolates analysed. Other less predominant genera included *Staphylococcus *(21.7%) and *Proteus *(8.7%).

**Table 2:** Bacterial genera identified by 16S rRNA gene sequencing of isolates from 10 prosthetic hip joints

Genus	Number of isolates (percentage)
*Leifsonia*	25 (54.3)
*Staphylococcus*	10 (21.7)
*Proteus*	4 (8.7)
*Brevundimonas*	3 (6.5)
*Salibacillus*	2 (4.3)
*Methylobacterium*	1 (2.2)
*Zimmermannella*	1 (2.2)

The total number of samples was 46.

The bacterial isolates obtained and identified by 16S rRNA gene sequencing are categorised to species level in Table 3. The most prevalent species was *Leifsonia aquatica *(43.5%), followed by *Staphylococcus epidermidis *(19.6%) and *Leifsonia shinshuensis *(10.9%).

**Table 3:** Bacterial species identified by 16S rRNA gene sequencing of isolates from 10 prosthetic hip joints

Species	Number of isolates (percentage)
*Leifsonia aquatica*	20 (43.5)
*Staphylococcus epidermidis*	9 (19.6)
*Leifsonia shinshuensis*	5 (10.9)
*Proteus mirabilis*	4 (8.7)
*Brevundimonas *sp. V4.BO.05	3 (6.5)
*Salibacillus *sp. YIM-kkny 16	2 (4.3)
*Methylobacterium radiotolerans*	1 (2.2)
*Staphylococcus pasteuri*	1 (2.2)
*Zimmermannella alba*	1 (2.2)

The total number of samples was 46.

### Culture-independent methods

A total of 512 clones from the five clinically infected and the five clinically non-infected prosthetic hip joints were subjected to restriction enzyme analysis. Because many RFLP groups contained multiple clones with the same restriction profiles, a single representative clone from each group was sequenced. A DNA sequence of at least 500 base pairs was obtained for each clone. In all, 118 clones were sequenced.

The bacterial genera/groups identified across the 10 samples are shown in Table 4. *Lysobacter *was the most prevalent genus, accounting for over 60% of the clones analysed. Other bacterial genera/groups identified included gamma proteobacterium (8.0%), *Stenotrophomonas *(6.6%), *Methylobacterium *(4.7%) and *Staphylococcus *(4.7%). The bacterial species identified in the 10 samples are shown in Table 5. *Lysobacter enzymogenes *was the most prevalent species (31.4% of analysed clones), followed by *Lysobacter *sp. C3 (28.3%), gamma proteobacterium (6.6%), *Methylobacterium *SM4 (4.7%) and *Staphylococcus epidermidis *(4.7%). A total of 28 phylotypes were identified.

**Table 4:** Bacterial genera/groups identified by 16S rRNA gene sequencing of clones from 10 prosthetic hip joints

Genus	Number of clones analysed (percentage)	Number of clones sequenced (percentage)
*Lysobacter*	312 (60.9)	52 (44.1)
Gamma proteobacterium	41 (8.0)	8 (6.8)
*Stenotrophomonas*	34 (6.6)	9 (7.6)
*Methylobacterium*	24 (4.7)	5 (4.2)
*Staphylococcus*	24 (4.7)	5 (4.2)
Various bacterial clones	23 (4.5)	10 (8.5)
*Proteus*	18 (3.5)	5 (4.2)
*Bradyrhizobium*	11 (2.1)	4 (3.4)
*Bacteroides*	6 (1.2)	3 (2.5)
Hydrothermal vent eubacterium	6 (1.2)	6 (5.1)
Iron-oxidising lithotroph ES-1	5 (1.0)	5 (4.2)
Methylobacteriaceae^a^	4 (0.8)	2 (1.7)
*Acidobacteria*	1 (0.2)	1 (0.8)
*Eubacterium*	1 (0.2)	1 (0.8)
Endophytic bacterium	1 (0.2)	1 (0.8)
*Xylella*	1 (0.2)	1 (0.8)

In all, 512 clones were analysed, and 118 clones were sequenced.^a^Family.

**Table 5:** Bacterial species identified by 16S rRNA gene sequencing of clones from 10 prosthetic hip joints

Species	Number of clones analysed (percentage)	Number of clones sequenced (percentage)
*Lysobacter enzymogenes*	161 (31.4)	27 (22.9)
*Lysobacter *sp. C3	145 (28.3)	24 (20.3)
Gamma proteobacterium N4-7	34 (6.6)	7 (5.9)
*Methylobacterium *SM4	24 (4.7)	5 (4.2)
*Staphylococcus epidermidis*	24 (4.7)	5 (4.2)
Uncultured bacterium clone mw5	19 (3.7)	6 (5.1)
*Proteus mirabilis*	18 (3.5)	5 (4.2)
*Stenotrophomonas *sp. SAFR-173	18 (3.5)	7 (5.9)
*Stenotrophomonas maltophilia*	16 (3.1)	2 (1.7)
*Bradyrhizobium *sp. BC-C1	8 (1.6)	1 (0.8)
Uncultured gamma proteobacterium clone B22B17	7 (1.4)	1 (0.8)
*Bacteroides fragilis*	6 (1.2)	3 (2.5)
*Lysobacter *sp. IB-9374	6 (1.2)	1 (0.8)
Hydrothermal vent eubacterium	6 (1.2)	6 (5.1)
Iron-oxidising lithotroph ES-1	5 (1.0)	5 (4.2)
Uncultured Methylobacteriaceae clone M3Ba28	2 (0.4)	1 (0.8)
Uncultured Methylobacteriaceae clone 10-3Ba12	2 (0.4)	1 (0.8)
*Bradyrhizobium japonicum*	1 (0.2)	1 (0.8)
*Bradyrhizobium *sp. CCBAU	1 (0.2)	1 (0.8)
Uncultured rape rhizosphere bacterium wr0008	1 (0.2)	1 (0.8)
*Acidobacterium *sp. TAA166	1 (0.2)	1 (0.8)
Endophytic bacterium	1 (0.2)	1 (0.8)
*Xylella fastidiosa*	1 (0.2)	1 (0.8)
Uncultured *Eubacterium *clone GL178.11	1 (0.2)	1 (0.8)
Uncultured bacterium Br-z43	1 (0.2)	1 (0.8)
Uncultured bacterium clone BA017	1 (0.2)	1 (0.8)
Uncultured bacterium clone LG25	1 (0.2)	1 (0.8)
Uncultured bacterium clone I-9	1 (0.2)	1 (0.8)

In all, 512 clones were analysed, and 118 clones were sequenced.

Thirty-six (7.0%) analysed clones represented 10 different uncultured phylotypes (Table 6). The most prevalent phylotype was uncultured bacterium clone mw5, representing 19 (3.7%) of the clones analysed. No potentially novel species (sequence identities less than 98%) were identified.

**Table 6:** Details of clones sequenced representing uncultured species

Sample no. (clone)	Sequenced bases available for BLAST	Matching bases	Sequence identity (percentage)	Accession no.	Identified bacterial species
4 (32)	621	542/550	98.5	AF323759	Uncultured bacterial clone BA017
6 (21)	513	494/503	98.2	AY038628	Uncultured *Eubacterium *clone GL178.11
24 (32)	683	651/658	98.9	AJ295469	Uncultured rape rhizosphere bacterium wr0008
32 (24)	527	469/479	97.9	AY360534	Uncultured Methylobacteriaceae clone 10-3Ba12
32 (32)	654	626/632	99.1	AY625143	Uncultured bacterial clone I-9
34 (29)	570	535/543	98.5	AY539816	Uncultured gamma proteobacterium clone B22B17
42 (19)	733	722/731	98.8	AY360692	Uncultured Methylobacteriaceae clone M3Ba28
47 (21)^a^	510	467/477	97.9	DQ163946	Uncultured bacterium clone mw5
58 (24)	621	567/576	98.4	AF507008	Uncultured bacterium Br-z43
87 (28)	692	628/637	98.6	AY977912	Uncultured bacterium clone LG25

^a^Six clones possessed identical RFLP profiles.

## Discussion

The risk of infection after hip replacement surgery remains unacceptably high. A greater understanding of which microorganisms may be involved in the infective process will be necessary for an improvement in infection rates and subsequently an improvement in treatment methods. In addition to the uncertainty over the true prevalence of prosthetic hip joint infection, in many cases there is also debate over the source of the infection. The skin microbiota of hospital staff or patients has been assumed to be a likely reservoir of infection. For some patients it has been demonstrated that the oral cavity is the source of prosthetic joint infection [14-17]. However, there is continuing debate over the need for antibiotic prophylaxis when patients with joint prostheses undergo dental treatment procedures that stimulate a bacteraemia [18].

Previous studies have shown that PCR amplification of the 16S rRNA gene, a highly conserved region within the bacterial genome, is invaluable in the detection of the bacterial types involved in prosthetic hip joint infections [10,19,20]. However, it has been claimed that PCR assays cannot be used to identify each pathogen in cases of mixed infection [21] and havepoor positive predictive value for hip joint infection [22]. However, we have shown in the present study that gene amplification and sequencing of 16S rRNA is useful in identifying single bacterial species isolated by standard culture techniques as well as in defining the mixed bacterial flora found on the surface of the prosthetic hip joints by using a direct PCR and sequencing approach. It is important to note that the necessary precautions were taken to avoid contamination in the clinical and laboratory settings. The prosthetic hip samples were collected by a surgical team wearing body exhaust suits in an operating theatre with a clean-air enclosure and were packaged in sterile bags. In addition, PCR was performed under stringent conditions and with the use of appropriate controls to prevent false-positive results. Processing of the prosthetic hip joints and subsequent DNA extractions were conducted in a separate laboratory from the PCR assays. All of the reagents for PCR were also stored separately from the positive DNA samples, with the reagents being aliquoted before use to avoid contamination. Finally, a negative control was included with each PCR assay to rule out possible contamination by bacterial DNA. A sterile hip, autoclaved and processed in an identical manner to the 10 test hip joint components, yielded a negative PCR result.

Clinical diagnosis of the 10 cases studied identified five hip replacement devices removed because of bacterial infection and the other five because of aseptic loosening. Comparison of the bacterial species identified in both clinical situations showed a large number of species that may be involved in infection. The microflora associated with each prosthetic hip joint studied was very similar, irrespective of the clinical reason for prosthesis removal. No specific bacterial species that can be associated with clinical infection or aseptic loosening were found. The bacterial species identified take the form of a biofilm attached to the surface of the removed prosthesis in both infected and non-infected cases. It may be that one organism alone or several bacterial species have a role in initiating, prolonging or activating simultaneous or subsequent joint infections. They may also have a role in rendering the joint more susceptible to clinical infections.

The predominant bacteria identified by culture-independent methods from the surface of all the prosthetic hip joints (both infected and non-infected cases) were *Lysobacter enzymogenes *and *Lysobacter *sp. C3. Other members of the *Lysobacter *clade [23] identified were *Lysobacter *sp. IB-9374, iron-oxidising lithotroph ES-1 and hydrothermal vent eubacterium, in addition to the closely related species *Stenotrophomonas maltophilia*. These species, which have not previously been reported to be involved in prosthetic hip infection, were not identified by standard culture techniques. The role of *Lysobacter*-type species in prosthetic hip joint infections is unknown and further research will be required to study the virulence factors involved in infection and the effects on the human immune system. However, *Lysobacter*-type species have been shown to be important pathogens in hospital-acquired infections [24]. In fact, it has recently been demonstrated that various *Lysobacter*-type species have the ability to form biofilms readily on various substrates. These include *Stenotrophomonas maltophilia*, *Xylella fastidiosa *and *Xanthomonas axonopodis *[25-27]. It is therefore perhaps unsurprising that these species were identified on the prostheses of the patients in our study.

*S. maltophilia *was first reported as an environmental species but is now known to be an emerging hospital-acquired pathogen that, among others, has been isolated from gentamicin-loaded polymethylmethacrylate beads in orthopaedic revision surgery [28]. A previous report of a strain of this organism, which was positively charged, demonstrated favourable adhesion kinetics to surfaces such as glass and Teflon [29], and *S. maltophilia *is now known to adhere avidly to medical implants and catheters to form a biofilm [30]. Furthermore, the organism has been implicated in a range of human infections, including septic arthritis in a patient with AIDS [31].

Sullivan and colleagues [23] described the evolutionary relationship between members of the *Lysobacter *clade. *Lysobacter *sp. strain C3 was initially identified as *Stenotrophomonas maltophilia *[32]. *S. maltophilia *has been identified by 16S rRNA gene sequencing as the predominant species in advanced noma lesions [33] and has been isolated from a case of acute necrotising gingivitis in an immunocompromised individual [34]. Whether *Stenotrophomonas*/*Lysobacter *species are natural members of the oral flora or are merely transient would require further study. However, a high oral carriage of *S. maltophilia *in a Tibetan population has been reported [35].

From the cultured bacterial isolates sequenced, nine different species were identified that are thought to be involved in prosthetic hip joint infections; *Staphylococcus *species have previously been described in this context [7,20,22], but the other bacteria identified are not commonly associated with prosthetic hip joint infections. *Leifsonia *species are known to favour moist environments, and in association with other bacterial species they cause infections of the central venous catheter used as vascular access for haemodialysis [36]. *Proteus mirabilis *has been described in joint infections [20] but is more commonly associated with urinary tract infections [37]. *Brevundimonas *species are rarely isolated from clinical samples; the role of this species in human disease needs further research, but it has been associated with two cases of bloodstream infections [38]. *Zimmermannella alba *has been isolated from human blood [39] but has not been reported to be involved in prosthetic hip joint infections. *Methylobacterium radiotolerans *[40] and *Salibacillus *species (GenEMBL accession number AY121439) are environmental bacteria more commonly found in plants and salt water lakes, respectively.

A further 21 species of bacteria were identified by culture-independent methods. As stated previously, *Staphylococcus *and *Proteus *[7,20,22] species are associated with prosthetic hip joint infections and were isolated by microbiological culture and culture-independent methods in the current study. Several other species that differ from those identified by microbiological culture were identified by culture-independent methods. *Bacteroides fragilis *has previously been associated with hip joint infections and has been isolated in cases of septic arthritis [41]. Many of the other species identified have been more commonly isolated from plants and soil; these include gamma proteobacterium [23], *Methylobacterium *[42], *Bradyrhizobium *[43], *Acidobacteria *[44] and *Xyella *[45]. For example, *Xyella fastidiosa *is a phytopathogenic bacterium responsible for diseases in many economically important crops [45]. The uncultivable species identified in the present study are environmental bacteria [46,47]. Although these bacteria could not be cultured by standard microbiological techniques in the present study, this might have been due to the fastidious growth requirements of these organisms, or to the fact that bacteria growing within a surface-associated biofilm displayed viable but non-culturable tendencies. A recent review has highlighted the plethora of clinical and environmental bacteria that have this characteristic, but whether they are capable of pathogenic traits has yet to be determined [48]. In addition, the use of prophylactic antibiotics during the surgical procedure would hinder the ability to culture and isolate bacteria. However, in our current study none of the patients received antibiotic prophylaxis before surgery.

It was interesting to note that the bacterial species found in the preoperative/perioperative samples by culture (coagulase-negative *Staphylococcus*, patients 3 and 6; *P. mirabilis*, patient 7) were also found on the surface of the corresponding prosthetic hip joints by both culture and culture-independent methods. This is suggestive of potential involvement of these species in the infective process. Bacteria were cultured from preoperative/perioperative samples in only 3 out of 10 cases analysed, whereas all 10 corresponding prosthetic hip joints were positive for the presence of bacteria by both culture and culture-independent methods. Furthermore, bacteria were isolated from preoperative/perioperative samples in only three of the five cases classified as being clinically infected. This clearly suggests that standard methods for determining infection in these cases are unreliable.

Several unusual bacterial species have been isolated during this study that have not previously been described as human pathogens and have not been implicated in human infections of prosthetic hip joints. Most of the unusual species identified are environmental bacteria isolated more commonly from plants. Further research is required into the pathogenicity of these bacterial species. It may be that they show pathogenic potential only when they are part of a biofilm in association with other bacterial species. This is indeed the case with *Leifsonia *species, which are reported to cause infections of the central venous catheter when they are in a biofilm with two other unusual bacterial species [36]. Furthermore, Duan and colleagues [49] demonstrated that key virulence factors from the biofilm-forming organism *Pseudomonas aeruginosa *were upregulated in the presence of oropharyngeal commensal flora. These studies indicate the key role of polymicrobial biofilms in clinical biofilm diseases, and how cell-cell interactions from non-pathogenic organisms may promote the progression of disease.

There were differences in the bacterial species identified by the microbiological culture and culture-independent methods used in the current study. The species identified that were common to both methods were from the genera *Staphylococcus *and *Proteus*, which have previously been associated with hip joint infections. One possible reason for this is the culture techniques used: in this study we used standard culture media and incubation conditions, which broadly enabled us to maximise the culture of bacteria. However, this approach may not have been specific enough for other fastidious organisms. Because the species present on the surface of the prosthetic hip joints are unknown, it is not possible to use specialised media and conditions at this stage, primarily because of cost implications and the time required to process specimens on a vast array of media. Now that it is known that *Lysobacter*-type species are predominant in prosthetic hip joint infections it will be possible to use specialised media to culture them from the hip sonicate. This exemplifies the validity for culture-independent methods to be conducted in parallel with the culture techniques, so as to identify the entire array of infecting bacteria in each clinical sample.

Another reason for the differences observed may be that primer bias occurs during the PCR procedure. Primer bias results in the unequal amplification of PCR products, resulting in distortion in the product numbers for each bacterial type. PCR primer bias is thought to be caused by inhibition of amplification by self-annealing of the most abundant templates in the late stages of amplification [50] or as a result of differences in the amplification efficiency of templates [51]. We have shown that neither method on its own can isolate all bacteria involved in prosthetic hip joint infections. The vast majority of the bacteria identified that had previously been characterised were Gram-negative species, with the only Gram-positive species being *Staphylococcus epidermidis*, *Staphylococcus pasteuri*, *Leifsonia aquatica*, *Leifsonia shinshuensis*, *Salibacillus *sp. and *Zimmermannella alba*. Some studies, which used 16S rRNA gene sequencing to identify bacteria in a relatively small number of clinical specimens, adopted the approach of sequencing about 50 clones from each library generated per sample [33,52]. Because of the relatively large number of samples analysed in our study we sought to minimise the sequencing of identical clones by screening with RFLP analysis, and sequencing a single representative clone from each RFLP group. This approach has been used successfully in many studies to avoid sequencing redundancy and to estimate bacterial diversity within clinical specimens [53-55].

From our findings it can be seen that a wide range of bacteria are potentially associated with prosthetic hip joint infections. Further research is required to identify other bacteria involved in infections, because other species have been reported in the literature that were not identified in this study. The knowledge of the bacteria involved in infection can further our research into biofilm formation, into the signalling patterns between the bacteria within the biofilm and into the effects on the human immune system of these infecting pathogens that lead to prosthetic hip joint infections. Although the immediate clinical significance of the present study is somewhat limited, it nonetheless represents a useful preliminary investigation into the microbiology of the aseptic loosening and infection of prosthetic hip joints. Ideally, the study should be expanded to include a larger number of specimens to determine whether true differences exist with regard to the microflora associated with both groups. Because the present study detected only microbial DNA, it is unknown which of the bacteria identified were viable. This could be overcome in future studies by the detection of bacterial mRNA rather than DNA, which would identify only the transcriptionally active (viable) bacteria present on infected and uninfected prosthetic hip joints. This would give a greater insight into which species may be of clinical significance. Ultimately, such data could inform both antibiotic usage in prosthetic joint surgery (both prophylactic and therapeutic) and the relative merits of one-stage and two-stage revisions. However, it should be noted that the antibiotic susceptibility profiles of the bacterial species identified by culture-independent techniques cannot be determined, and this may hinder the development of improved antimicrobial therapy regimes. This study may also have a potential impact on improving the laboratory diagnosis of prosthetic hip joint infections.

## Conclusion

A wide range of bacteria can be found on the surface of prosthetic hip joints removed at revision arthroplasty. No significant differences were observed in the microflora associated with infected and non-infected cases. However, the predominant species were members of the *Lysobacter *genus.

## Abbreviations

FAA = fastidious anaerobe agar; PCR = polymerase chain reaction; RFLP = restriction fragment length polymorphism; THA = total hip arthroplasty.

## Competing interests

The authors declare that they have no competing interests.

## Authors' contributions

KED planned and performed the work and helped to draft the manuscript. MPR participated in study design, planned the work and helped to draft the manuscript. AL provided technical support. VEH developed some of the methodology. GR and JB participated in the study design. DA coordinated sample collection. All authors read and approved the final manuscript.
